# Psychometric properties of the Arabic version of the Basic Psychological Needs Satisfaction-Frustration Scale (BPNSFS)

**DOI:** 10.1186/s40359-020-00506-1

**Published:** 2021-01-26

**Authors:** Kashef N. Zayed, Ehab N. Omara, Nasser Y. al-Rawahi, Ali K. al-Shamli, Asma K. al-Attiyah, Ahmad A. al-Haramleh, Mahmoud S. Azab, Ghada M. al-Khasawneh, Mohammed A. Hassan

**Affiliations:** Department of Physical Education and Sport Sciences, College of Education, Sultan Qaboos University, Muscat, Oman

**Keywords:** Basic psychological needs, Self-determination theory, Mental well-being, Depression

## Abstract

**Background:**

The aim of this study is to validate the Arabic version of the Basic Psychological Need Satisfaction and Frustration Scale (BPNSFS), and to investigate the extent of its invariance across five Arab countries and gender.

**Methods:**

A back-translated version of the BPNSFS, the second version of the Beck Depression Inventory (BDI-II), and the Warwick-Edinburgh Mental Well-Being Scale (WEMWBS) were administered to a sample consisting of 1082 undergraduate students affiliated with universities in five Arab countries (487 males and 595 females: M_age_ = 20.04 ± 1.87 years). The data of the BPNSFS were examined for univariate and multivariate normality using Shapiro–Wilk tests and Mardia’s coefficient, respectively. To evaluate and compare the four models with confirmatory factor analysis (CFA), we used the following goodness-of-fit indices: the chi-square value (χ^2^), comparative fit index (CFI), Tucker-Lewis index (TLI), Root Mean-Square Error of Approximation (RMSEA), and Baysian Information Criterion (BIC). A multi-group CFA [Byrne in Structural equation modeling with EQS: basic concepts, applications, and programming, Routledge, Abingdon, 2013] on the BPNSFS structure to examine its invariance across the five Arab countries and across genders.

**Results:**

The results of confirmatory factor analysis supported the generalizability of the BPNSFS’s six-factor model to the five Arab countries. The relationships between the six psychological needs satisfaction and frustrations and both mental health and symptoms of depression provide additional evidence on the construct validity of the BPNSFS through cross cultural data. The findings of BPNSFS’s measurement invariance across males and females and across the five Arab countries help ensure that the latent means are comparable across these different groups.

**Conclusions:**

The study concluded that the Arabic version of the BPNSFS which measures satisfaction and frustration of the three basic needs (autonomy, competency, and relatedness) is proved to be invariant across the five Arab countries and gender and can be used to compare the basic psychological needs in the Arab context.

## Background

The term "need" in our everyday language refers to the desire to have something that an individual particularly prefers. Various evidence from different cultures support the relationship between fulfillment of the basic psychological needs and mental health, such as vitality [32], good mood [22] and low anxiety [5, 6]. Furthermore, studies showed that failure to satisfy the basic psychological needs is associated with mental illness [e.g., 12, 15].

This study aims to validate the Arabic version of the Basic Psychological Needs Satisfaction and Frustration Scale (BPNSFS) which was developed by Chen, et al. [3] as well as examining its’ contrast validity through examining the relationship between satisfaction and frustration of basic psychological needs, and mental well-being and self-reported depressive symptoms.

### Basic psychological needs

The Basic Psychological Needs Theory (BPNT), which is one of the main sub-theories from the SDT, assumed that individuals have three basic needs: autonomy, competence, and relatedness. The term “autonomy” is based on people being empowered when they feel they have freedom to choose not to be controlled by others. Thus, autonomy satisfaction means that behavior is self-determined and in accordance with individual's will and desire, consequently, it is accompanied by a sense of fulfillment and pleasure [23]. In contrast, autonomy frustration is expected to lead to a sense of feeling controlled and forced to behave in a certain way and therefore leads to an unpleasant experience [26, 33]. The term “competence” refers to the individuals’ need to feel efficient and in control. Hence, competence satisfaction is seen as a sense of effectiveness and capability to achieve goals, while competence frustration leads to a sense of failure and doubting own capabilities [25]. The “relatedness” term refers to the inherent human needs as social beings seek to be contacted to others and in a good relationship with them. Thus, relatedness satisfaction refers to connecting with others positively and feeling attached to them [29]. Relatedness frustration, however, describes feelings of isolation, loneliness and social failure [34].

### Mental well-being and ill-being

Mental well-being describes the psychological efficacy of the individual, subjective experience of happiness and satisfaction with life, on one hand, and the ability to develop and maintain good and stable relationships with others, on the other hand [31]. It includes the extent of the individual's ability to manage his life affairs and self-acceptance and appreciation. The achievement of mental well-being is more than just prevention of mental disorders. In contrast to mental well-being mental ill-being refers to variety of mental disorders which negatively affect the mood and limit the ability to work effectively and appropriately in different aspects of life [31]. It includes a wide range of mental health conditions that affect mood, thinking, and behavior, including depression, anxiety disorders and others [17]. In this study, we considered depressive symptoms that may affect individuals at certain times in their lives, as indicators to the existence of mental illness.

### The effect of basic psychological needs satisfaction or frustration on well-being and ill-being

Studies have revealed that satisfying these needs is important and varies according to individual and cultural determinants [26] and concluded that need satisfaction is associated with mental well-being and the highest levels of motivation [25], while need frustration is associated with mental ill-being and low levels of motivation [20]. Previous studies conducted worldwide have sought to assess need satisfaction or frustration using different tools, such as the Basic Psychological Needs Satisfaction and Frustration Scale (BPNSFS). It has been implemented on a sample consisting of 1051 participants (Mean_age_ = 20 years) from four countries (China, Belgium, USA, and Peru). They found that satisfying the three basic psychological needs were positively correlated with life satisfaction and vitality [22] while the frustration of satisfying those needs were positively correlated with depressive symptoms [5]. These results are equivalent across the four countries. For this, it can be concluded that satisfying the three basic needs is an essential nutrient for achieving an optimal level of functioning across the different cultures and individuals as well. Later on, BPNSFS has been adapted and validated to different environments, such as in general and in work environments and in many domains targeting different groups of people including students, employees, people with disabilities, and athletes [31].

Cordeiro et al. [6] targeted a sample of undergraduate Portuguese students and found that the dimensions of satisfaction and frustration predict the mental well-being and ill-being of the participant. It was further carved out, that the six-factor solution best fitted the BPNSFS. In Italy, a study carried out by Costa et al. [7] retrieved the results that the following six factors of the scale are the most fit for the data, namely: autonomy satisfaction, autonomy frustration, competency satisfaction, competency frustration, relatedness satisfaction, and relatedness frustration. They also found that the structure of BPNSFS is invariance across gender and has proper degrees of validity and reliability that make it suitable instrument to investigate the satisfaction and frustration of the three basic psychological needs in the Italian environment.

Taken together, we can conclude from the previous studies which were conducted in different environments and dealt with various samples that the satisfaction of the three basic psychological needs (autonomy, competence, and relatedness) is directly and positively correlated to well-being and that the three needs’ frustration leads to ill-being. Also, previous studies that used BPNSFS provided strong evidence supporting the following six factors of the scale: autonomy satisfaction, autonomy frustration, competency satisfaction, competency frustration, relatedness satisfaction, and relatedness frustration, are different, but related to each other, whereas low satisfaction with any of the three basic psychological needs, such as feeling low competence will inhibit individual’s well-being, but competence frustration will generate ill-being or depressive symptoms [2, 7]. Finally, due to limited prior research on this topic in the Arab region that mainly investigates the effects of satisfying or frustrating of the basic needs, on well-being or ill-being, therefore it seems that there is an urgent need to conduct this study which is expected to contribute to the enriching of knowledge through providing relevant information from the Arab's culture, also, this study will introduce an Arabic validated version of BPNSFS for future uses in the Arab context.

### Objectives

The current study seeks to achieve two main objectives:

1. To validate the Arabic version of the Basic Psychological Need Satisfaction and Frustration Scale (BPNSFS).
2. To investigate the BPNSFS’s measurement invariance across the five Arab countries and gender which is required to make meaningful comparisons between the factor latent means across these groups.

## Methods

### Participants

The study targeted available classes in each of the participating universities, and after arranging with the lecturers, one of the authors or research assistants explained to the students in each class the nature of the study and informed them that participation is voluntary and anonymous. Accordingly,

1082 undergraduate students (487 males and 595 females; Mean age = 20.04 ± 1.87 years) voluntarily agreed to take part in our study, and all were undergraduate students registered in selected universities in five Arab countries: Oman (138), Saudi Arabia (237), Palestine (240), Qatar (233), and Jordan (234).

## Materials

### Demographic variables questionnaire

The participants were requested to answer demographic questions regarding their gender, age, university, and specialization.

### Basic Psychological Need Satisfaction-Frustration Scale (BPNSFS) (Additional File 1)

The original English version of the scale was back-translated into Arabic (“Appendix 1”) following the guidance of the second edition of the International Test Commission guidance for translating and adapting tests [14]. In addition, the first author of this study, two academics participated in the translation process, one of whom is bilingual. All appropriate modifications have been made until the content of the back translated version proved to be identical with the original English version of the scale. The scale consists of two parts: the first 12 items relate to basic need satisfaction, and the second 12 items relate to basic need frustration. Each of the two parts consists of three dimensions: Autonomy (satisfaction and frustration), relatedness (satisfaction and frustration), and competence (satisfaction and frustration). Thus, the scale consists of 24 items representing 6 dimensions (4 items each). Official approval was obtained from the authors before the scale was back-translated into Arabic. The reliability of the translated Arabic version using test–retest over a 2-week period indicated that scale reliability was reached (r = 0.74).

### Warwick–Edinburgh Mental Well-Being Scale (WEMWBS) (Additional File 2)

This scale was developed from a team of researchers from Scottish universities [30]. It measures mental well-being and consists of 14 positive items (ex. “I feel optimistic about the future”), and the respondent must answer each item according to a 5-level Likert scale (never happens = 1, rarely happens = 2, not sure = 3, often happens = 4, always happens = 5). Therefore, the minimum score is 14, and the upper limit is 70. The closer the sum score is to the minimum, the lower one’s mental well-being is, and vice versa. The scale was first translated to Arabic by Zayed [35] following the translation and adaptation recommendations of the International Test Commission [13]. The internal consistency reached 0.91 [35], while the reliability for the scale using test–retest method was 0.79, obtained with a restricted group from the original sample with a 14-day interval between the two tests’ administrations.

### Beck Depression Inventory-II (BDI-II) (Additional File 3)

The scale is considered one of the best methods of self-assessment and is widely used to measure depressive symptoms. It consists of 21 groups of items, each representing a particular symptom of depression: (0) illustrates that no depressive symptom is present, (1) indicates the presence of the symptom at a mild level, (2) indicates the presence of the symptom at a medium level, and (3) indicates the presence of the symptom at a severe level. In a meta-analysis evaluation through 25 years, the internal consistency of the scale ranged between 0.73 and 0.92, with a mean of 0.86 [1]. The internal consistency of the scale ranges between 0.73 and 0.92, with a mean of 0.86 [1]. The scale was standardized in Arab culture by Ghareeb [11], and in the Omani culture, the internal consistency of the scale ranged between 0.761 and 0.88 [35].

### Procedures

Participants in the study were recruited from several Arab universities, where they agreed to participate in the study. Paper questionnaires were distributed to them, which include demographic data and the other three tools (BPNSFS, WEMWBS, and BDI-II). The response period took about 15–20 min. After collecting the test and the exclusion of incomplete tests, the data were statistical analyzed.

### Statistical analysis

Data analyses were performed using SPSS (Version 23) and AMOS package (version 3.5.1, 2018). The data of the BPNSFS were examined for univariate and multivariate normality using Shapiro–Wilk tests and Mardia’s coefficient, respectively. To evaluate and compare the four models with confirmatory factor analysis (CFA), we used the following goodness-of-fit indices: the chi-square value (χ2), comparative fit index (CFI), Tucker-Lewis index (TLI), Root Mean-Square Error of Approximation (RMSEA), and Baysian Information Criterion (BIC). The model fit followed the cut-off values of less than 0.08 for the RMSEA and 0.90 or above for the CFI and TLI [17, 28]. Descriptive statistical analysis was performed to determine the mean, standard deviation, and correlation coefficients among the basic factors of the BPNSFS. For comparability purposes, tests for measurement invariance were conducted across the culture and across genders.

In a second step, we performed a multi-group CFA [2] on the BPNSFS structure to examine its invariance across the five Arab countries and across genders. A sequential model testing approach was conducted with the “lavaan” application in the R platform. Configural invariance addresses the equivalence of the factorial structure in different groups. Metric invariance tests the consistency of the strengths of the relations between the BPNSFS items and their respective underlying constructs across groups. Under metric invariance, the factor loadings are set to be equal across the study groups [8]. Thereafter, scalar or strong invariance were tested from constraining item intercepts to be equivalent across groups. This level-measurement invariance is necessary to compare the latent means among the study groups [21]. Form model comparisons, we used ΔCFI and ΔRMSEA which considered in acceptable levels when (ΔCFI < 0.01 and ΔRMSEA < 0.015; Cheung and Rensvold [4]. Finally, based on full scalar invariance as a baseline, we compared latent means across gender and countries. Critical Ratios (CR) were calculated to test whether the latent means are significantly different across groups. CR values larger than 1.96 and 2.58 indicate statistically significant differences in the latent means at 0.05 and 0.01 levels respectively.

## Results

### Factorial structure of BPNSFS

Initially, the Shapiro–Wilk test for univariate normality showed that the distribution deviated significantly from a normal distribution (the Shapiro–Wilk statistics ranged from 0.646–0.913, *p* < 0.001), and according to Mardia's multivariate coefficient for both skewness (7965.215) and kurtosis (59.475), the multivariate normality of the item responses did not hold (*p* < 0.001). Therefore, we used confirmatory factor analysis with the robust maximum likelihood method and correction to estimate the model parameters [27]. Confirmatory factor analysis was conducted to investigate the following four hypothetical models: (a) a two-factor model (need satisfaction and frustration), (b) a three-factor model (autonomy, relatedness, and competence, including both need satisfaction and frustration as reverse concepts), (c) a six-factor model (need satisfaction and frustration for autonomy, relatedness, and competence), and (d) a higher-order factor model. Table 1 summarizes the goodness-of-fit indices for the four models. Via comparison of the fit indices, initial estimation of the 6-factor model and higher-order factor model yielded a good fit to the data. According to BIC index, the 6-factor model slightly fits the data better than the higher order model: BIC_6-factors_ = 1000.09 < BIC_Higher-order_ = 1068.40.

**Table 1:** Model fit indices of four hypothetical models

Model (24 items)	$\chi^{2}$	*df*	*p*	CFI	TLI	RMSEA	90% CI for RMSEA	SRMR	BIC
Two-factor model	1008.64	251	0.000	0.874	0.862	0.053	0.049–0.056	0.053	1350.98
Three-factor model	2287.64	249	0.000	0.662	0.625	0. 087	0.084–0.090	0.091	2643.95
Six-factor model	559.94	237	0.000	0.946	0.938	0.036	0.032–0.039	0.040	1000.09
Higher-order factor model	684.14	245	0.000	0.927	0.918	0.041	0.037–0.044	0.066	1068.40

The standardized factor loadings of the items ranged from 0.29–0.65, 0.44–0.71, 0.46–0.69, 0.32–0.74, 0.60–0.70, and 0.47–0.66 (*p* < 0.001) on autonomy satisfaction, autonomy frustration, relatedness satisfaction, relatedness frustration, competence satisfaction, and competence frustration, respectively (Table 2).

**Table 2:** Standardized factor loadings of confirmatory factor analysis for six-factor model

Item	SFL	95% CI	
		Low	High
Autonomy satisfaction			
I feel a sense of choice and freedom in the things I undertake	0.347	0.30	0.40
I feel that my decisions reflect what I truly want	0.547	0.50	0.60
I feel my choices express who I truly am	0.294	0.24	0.34
I feel I have been doing what truly interests me	0.654	0.60	0.70
Relatedness satisfaction			
I feel that the people I care about also care about me	0.461	0.41	0.51
I feel connected with people who care for me, and for whom I care	0.688	0.64	0.74
I feel close and connected with other people who are important to me	0.672	0.62	0.72
I experience a warm feeling with the people I spend time with	0.649	0.60	0.70
Competence satisfaction			
I feel confident that I can do things well	0.601	0.55	0.65
I feel capable at what I do	0.671	0.62	0.72
I feel competent to achieve my goals	0.699	0.65	0.75
I feel I can successfully complete difficult tasks	0.603	0.55	0.65
Autonomy frustration			
My daily activities feel like a chain of obligations	0.465	0.42	0.52
I feel pressured to do too many things	0.712	0.66	0.76
I feel forced to do many things I wouldn’t choose to do	0.611	0.56	0.66
Most of the things I do feel like “I have to”	0.444	0.39	0.49
Relatedness frustration			
I feel the relationships I have are just superficial	0.319	0.27	0.37
I have the impression that people I spend time with dislike me	0.739	0.69	0.79
I feel that people who are important to me are cold and distant towards me	0.632	0.58	0.68
I feel excluded from the group I want to belong to	0.653	0.60	0.70
Competence frustration			
I feel like a failure because of the mistakes I make	0.581	0.53	0.63
I feel insecure about my abilities	0.659	0.61	0.71
I feel disappointed with many of my performance	0.538	0.49	0.59
I have serious doubts about whether I can do things well	0.469	0.42	0.52

### The internal consistency and the relationships between the BPNSFS’ factors

As shown in Table 3, and according to the rules of thumb mentioned in George and Mallery [10], the values of Cronbach’s alpha coefficient indicate that the six subscales showed questionable to-acceptable internal consistency that ranged between 0.63 for autonomy satisfaction and 0.74 for competence satisfaction. The Cronbach’s alpha coefficients for both frustration and satisfaction were 0.816 and 0.818 respectively, which considered as a good indicator for the internal consistency on the level of the two main subscales. As expected, the correlation coefficients among the three need satisfactions were positive and ranged from 0.47 to 0.57 (*p* < 0.001). Similarly, the correlation coefficients among the three need frustrations were positive and ranged from 0.44 to 0.50 (*p* < 0.001). Furthermore, satisfaction of each need was negatively correlated with frustration of the corresponding need.

**Table 3:** Means and standard deviations and correlations among the six factors

Factor	M	SD	1	2	3	4	5	6
1-Autonomy satisfaction (AS)	3.70	0.77	**0.63**	0.47**	0.47**	− 0.06*	− 0.13**	− 0.004
2-Relatedness satisfaction (RS)	4.16	0.80		**0.71**	0.57**	− 0.13**	− 0.37**	− 0.15**
3-Competence satisfaction (CS)	3.98	0.80			**0.74**	− 0.16**	− 0.23**	− 0.07*
4-Autonomy frustration (AF)	2.78	0.87				**0.65**	0.47**	0.44**
5-Relatedness frustration (RF)	2.25	0.88					**0.66**	0.50**
6-Competence frustration (CF)	2.74	0.68						**0.65**

*Correlation is significant at the *p* < 0.05 levels

**Correlation is significant at the *p* < 0.01 levels. The diagonal bold values are the Cronbach’s alpha coefficient

The same pattern of relationships among the six factors appears in the five countries, with clear differences in the degree of correlation across these samples (Table 4). The highest correlation coefficients among the three need frustrations as well as among the three need satisfactions were in the Jordanian sample, while the values of the correlation coefficient decreased relatively in other countries. Moreover, the majority of correlation coefficients between the need frustration factors and the need satisfaction factors were low negative correlations as well as they appear close to each other in the five groups.

**Table 4:** The correlation matrix among the 6-factors for each country

Country	AS	RS	CS	AF	RF
OM					
RS	0.392**				
CS	0.320**	0.568**			
AF	0.082	− 0.200*	− 0.215*		
RF	− 0.165	− 0.425**	− 0.193*	0.252**	
CF	− 0.074	− 0.304**	− 0.249**	0.426**	0.495**
KSA					
RS	0.243**				
CS	0.277**	0.391**			
AF	− 0.206**	− 0.245**	− 0.346**		
RF	− 0.211**	− 0.515**	− 0.289**	0.444**	
CF	− 0.118	− 0.243**	− 0.136*	0.296**	0.429**
JO					
RS	0.726**				
CS	0.679**	0.727**			
AF	0.085	0.037	0.014		
RF	− 0.027	− 0.124	− 0.132*	0.654**	
CF	0.198**	0.126	0.176**	0.678**	0.637**
PAL					
RS	0.482**				
CS	0.512**	0.491**			
AF	− 0.180**	− 0.187**	− 0.184**		
RF	− 0.218**	− 0.504**	− 0.240**	0.364**	
CF	− 0.103	-.227**	− 0.112	0.230**	0.363**
QTR					
RS	0.239**				
CS	0.391**	0.361**			
AF	− 0.108	− 0.304**	− 0.266**		
RF	− 0.075	− 0.486**	− 0.291**	0.518**	
CF	− 0.087	− 0.378**	− 0.260**	0.564**	0.536**

Oman (OM); Kingdom of Saudi Arabia (KSA); Jordan (JO); Palestine (PAL); Qatar (QTR); Autonomy Satisfaction (AS); Relatedness Satisfaction (RS); Competency Satisfaction (CS); Autonomy Frustration (AF), Relatedness Frustration (RF); Competency Frustration (CF)

**Correlation is significant at the 0.01 level.

*Correlation is significant at the 0.05 level

### Construct validity of BPNSFS scale

To further validate the Basic Psychological Need Satisfaction-Frustration Scale and identify how need satisfaction and frustration are associated with mental health and depression, we examined the correlations between the BPNSFS and both of mental health and symptoms of depression. Table 5 reports the correlation coefficients between the BPNSFS and the two concurrent measures. The correlations reported in Table 5 indicated that the participants who reported higher levels of need satisfaction tend to show a higher level of mental health than those reported in lower levels of satisfaction (*r* = 0.37 to 0.51; total = 0.54, *p* < 0.01), at the same time they will be less likely to have depressive symptoms (*r* = − 0.18 to − 0.22; total = − 0.24, *p* < 0.01). In contrast, those participants with higher levels of need frustration will be more likely to have depressive symptoms than others (r = 0.19 to 0.23; total = 0.26, *p* < 0.01), and they will be further from mental health (r = − 0.13 to − 0.28; total = − 0.28, *p* < 0.01). Accordingly, those previous relationships conclusively prove the construct validity of BPNSFS in accordance with the relationship between basic needs satisfaction and mental health, in one hand, and basic needs frustration, and depressive symptoms on the other. This conclusion is consistent with what has been reached by many researchers in different parts of the world [e. g. 2, 7].

**Table 5:** Correlations among need satisfaction, frustration, mental health, and depression

Variable	Need satisfaction				Need frustration			
	AS	RS	CS	Total	AF	RF	CF	Total
Mental health	0.37**	0.45**	0.51**	0.54**	− 0.18**	− 0.22**	− 0.18**	− 0.24**
Depression	− 0.13**	− 0.28**	− 0.26**	− 0.28**	0.19**	0.23**	0.20**	0.26**

Autonomy Satisfaction (AS); Relatedness Satisfaction (RS); Competency Satisfaction (CS); Autonomy Frustration (AF), Relatedness Frustration (RF); Competency Frustration (CF)

**Correlation is significant at the *p* < 0.01 levels

### Measurement invariance of BPNSFS

After determining that the six-factor model was the best-fitting model for the data, we used it as a baseline model to test the measurement invariance of the BPNSFS across cultures and genders. Multi-group CFA comparisons were conducted for the Arabic version of the BPNSFS to examine the level of measurement invariance across the study subgroups. The series of comparisons were carried out using R (lavaan package), and the results are presented in Table 6.

**Table 6:** Measurement invariance of the BPNSFS across country and gender levels

Level of measurement invariance	Model fit indices					Model comparison indices			
		*df*	CFI	TLI	RMSEA	Δχ^2^	Δ*df*	ΔCFI	ΔRMSEA
Country									
C1: Configural	1619.19	1185	0.917	0.904	0.045	–	–	–	–
C2: Metric	1809.82	1257	0.895	0.885	0.049	190.63	72	− 0.022	0.004
C3: Scalar	1897.99	1301	0.889	0.882	0.05	88.17	44	− 0.006	0.001
Gender									
G1: Configural	734.71	474	0.946	0.937	0.035	–	–	–	–
G2: Metric	750.52	492	0.946	0.94	0.035	15.81	18	0	0
G3: Scalar	791.23	510	0.942	0.937	0.035	40.71	18	− 0.004	0

For configural invariance across the five Arab countries, the results indicated that the values of the chi-square/degrees-of-freedom ration, CFI, TLI, and RMSEA were within the acceptable range (χ^2^⁄df = 1.37, CFI = 0.917, TLI = 0.904, RMSEA = 0.045). These findings support the assumption that the BPNSFS construct is identical across cultures. Additionally, the results indicated that full metric invariance was adequately met, since the absolute value of ΔRMSEA < 0.015 with a slightly increase in ΔCFI.

Moreover, scalar invariance was tested from constraining the item intercepts to be equal across the five groups. The comparison between C3 and C2 showed that the full scalar invariance across countries was met (ΔCFI < 0.01 and ΔRMSEA < 0.015). These results could be useful for making the comparisons of latent means across the five Arab countries.

For gender invariance, the results indicated that the six-factor structure of the BPNSFS was the same for males and females (χ^2^⁄df = 1.55, CFI = 0.946, TLI = 0.937, RMSEA = 0.035). Additionally, the factor loadings were equivalent for the two groups (G2: ΔCFI < 0.001 and ΔRMSEA < 0.001), which supports the full metric invariance of the BPNSFS across genders. Moreover, by constraining the intercepts of the BPNSFS items to be equal for males and females, the fit indices supported full scalar invariance across genders (G3: ΔCFI = − 0.004 and ΔRMSEA < 0.001). The full scalar invariance of BPNSFS across gender can be considered as a baseline to test the differences between males and females in the latent means.

### Latent mean differences

Based on the verification of the scalar invariance across countries and gender, we can compare latent mean scores across these groups. The results of latent mean differences between males and females in BPNSFS’s factors showed that females had significantly higher AS, RS, and CS than males (CR_AS_ = 3.25, CR_RS_ = 5.35, CR_CS_ = 2.87; *p* < 0.01). Also, the females had significantly lower RF and CF than males (CR_RF_ = 5.23, CR_CF_ = 5.04; *p* < 0.01), and the both of them had the same level of AF (CR_AF_ = 0.79; *p* = 0.432). For the differences across countries, the results presented in Table 7 showed that the level of AS, RS, and CS in the Jordanian sample appears to be higher than the rest of the four countries. Also, the levels of CS and RS in the Qatari sample were significantly higher than in the other four countries. Moreover, the level of RF in the Qatari and Palestinian samples was significantly lower than in Jordan. In the Palestinian sample, the level of AF was the lowest compared with its levels in Saudi, Qatari, and Omani samples. Additionally, the levels of CF in Qatari and Palestinian samples were significantly lower than the levels in the other countries.

**Table 7:** Critical ratios for differences between latent means across countries

Factor	Country	JO	KSA	OM	PAL	QTR
AS	JO	(3.68)				
	KSA	2.53	(3.54)			
	OM	3.17	1.08	(3.68)		
	PAL	3.01	0.55	0.64	(3.71)	
	QTR	2.57	0.07	1.19	0.66	(3.74)
RS	JO	(4.12)				
	KSA	4.65	(3.82)			
	OM	4.14	0.08	(4.29)		
	PAL	3.58	1.17	1.05	(4.05)	
	QTR	7.36	3.79	2.93	4.68	(4.12)
CS	JO	(4.02)				
	KSA	3.99	(3.73)			
	OM	4.50	1.22	(4.26)		
	PAL	3.82	0.23	1.42	(4.01)	
	QTR	7.44	4.09	2.00	4.36	(4.12)
AF	JO	(2.91)				
	KSA	1.83	(2.79)			
	OM	3.14	1.65	(2.92)		
	PAL	0.61	2.48	3.68	(2.75)	
	QTR	1.88	0.04	1.62	2.54	(3.05)
RF	JO	(2.94)				
	KSA	1.88	(3.03)			
	OM	1.39	0.42	(2.87)		
	PAL	2.91	1.06	1.42	(2.89)	
	QTR	3.24	1.52	1.84	0.56	(2.96)
CF	JO	(2.15)				
	KSA	0.46	(2.19)			
	OM	0.86	0.48	(1.91)		
	PAL	2.51	2.15	1.33	(2.00)	
	QTR	3.67	3.37	2.32	1.18	(2.11)

The values between brackets are the latent means across countries and BPNSFS' factors

Oman (OM); Kingdom of Saudi Arabia (KSA); Jordan (JO); Palestine (PAL); Qatar (QTR); Autonomy Satisfaction (AS); Relatedness Satisfaction (RS); Competency Satisfaction (CS); Autonomy Frustration (AF), Relatedness Frustration (RF); Competency Frustration (CF)

## Discussion

Regarding the first objective of this study, which aimed to validate the Arabic version of the Basic Psychological Need Satisfaction and Frustration Scale (BPNSFS). To achieve the study objectives, we analyzed the internal consistency and utilized the Conformity Factor Analysis (CFA) to examine the validity of the scale’s six-factor structure. The results of the study found that the internal consistency obtained from the entire sample reached adequate values (Alpha = 0.818 and 0.816) for both main subscales: Needs satisfaction and needs frustration. Also, the establishment of scalar invariance (equal factor loading and intercepts) across both gender and countries indicated that the latent mean differences of the BPNSFS’s subscales can be compared directly as they reflect changes in true scores rather than measurement artifacts. This can be considered one of the main benefits of measurement’s invariance. In other words, the six latent factors that measure basic psychological needs are interpreted similarly across the study groups. These findings are consistent with what was reached by the original study [3] and also by many other studies across different cultures [e. g. 9, 19, 22]. Another clear evidence of the validation of the Arabic version of BPNSFS is its ability to distinguish between needs satisfaction and needs frustration: Needs satisfaction with the three basics needs is associated positively with mental well-being, while needs frustration is associated positively with mental ill-being. The study also approved that satisfaction with each need correlate with mental well-being, and that frustration with each need correlate to mental ill-being. In other words, the result indicated that need satisfaction has a positive effect on mental health and a negative effect on depression, whereas need frustration has a negative effect on mental health and a positive effect on depression. These results introduced strong evidence of the validity of the scale, on one hand, and emphasize the clear function of needs satisfaction and frustration in influencing the psychological stability of the individuals, on the other hand. As needs satisfaction contribute to mental well-being and needs frustration contribute to mental ill-being indictor (depressive symptoms), which represent the core premise of the BPNT. These correlations are also proven worldwide [e.g. 2, 6, 7, 22]confirming the universal rule of the BPNT across cultures.

Additionally, because the intercepts of the BPNSFS items were equivalent across the groups, multi-group comparisons of factor mean have become possible and meaningful. Therefore, we can be confident that any statistically significant differences in groups’ means are not due to differences in the BPNSFS properties of different groups. According to Milfont and Fischer [21], assessing configural, metric, and scalar invariance is sufficient for establishing measurement invariance of a scale. The results of measurement invariance in the current study support the validity of the BPNSFS and can be considered an indicator of the possibility of comparing basic psychological needs in the five Arab countries and for males and females. Additionally, one of the significant contributions of the current study is to demonstrate the consistency of the six-factor structure of basic psychological needs not only in the five Arab countries—Oman, Jordan, Qatar, Saudi Arabia, and Palestine—but also in Japan [22] and Portugal [6]. The latent mean differences between males and females indicated that females scored higher than males in autonomy satisfaction, relatedness satisfaction, and competence satisfaction, while they scored lower in relatedness frustration and competence frustration. These results can be understood in light of what has been reached by several studies that females outperform males academically [18], which would contribute to satisfying females’ competence need and at the same time enhancing their independency in making their academic decisions, which contributes to satisfying their autonomy need. With regard to females achieving higher in relatedness satisfaction, this result leads us to build assumption that participants females undergraduate seems to be more socially oriented compared to males’ counterparts, perhaps as a result of the lack of university activities opportunities available to them. This assumption needs farther investigations.

Also, the latent mean showed some significant differences across the five Arab countries. These differences could be referred to the differences in economic situation between the countries. Regardless of the causes of these differences, we can conclude that our findings supported the universal role of basic psychological need satisfaction and frustration across countries. These results. Our findings are in harmony with what have been reached by several studies conducted worldwide [e. g. 3, 22].

Several limitations should be noted in the current study. First, some item had a standardized factor loading lower than 0.4, which need to be discussed in a future validation study of BPNSFS in the Arab region. Also, factor variance and covariance invariance was not examined in the current study. Future studies should investigate the structural invariance to examine the construct validation of BPNSFS. Moreover, the establishment of the full invariance across countries was based on the difference in RMSEA with a slight increase in ΔCF. So, the replication of the study would be important to support the measurement invariance of BPNSFS across countries.

## Conclusion

The study concluded that the Arabic version of the BPNSFS which measures satisfaction and frustration of the three basic needs (autonomy, competency, and relatedness) is proved to be invariant and having proper psychometric specifications making it a reliable and valid instrument to assess basic psychological need satisfaction and frustration among undergraduate Arab students.

## Supplementary Information

**Additional file 1**. The Basic Psychological Need Satisfaction-Frustration Scale (BPNSFS).

**Additional file 2**. The Warwick-Edinburgh Mental Well-Being Scale (WEMWBS).

**Additional file 3**. Beck Depression Inventory-II (BDI-II).

## Appendix 1: The Arabic version of the “Basic Psychological Needs Satisfaction-Frustration Scale (BPNSFS)”

### النسخة العربية من مقياس الرضا – الإحباط عن تحقيق الحاجات النفسية الأساسية

**Table:**

العبارة	الرقم
أمتلك الحرية في اختيار ما أريد في حياتي	1
معظم الأفعال التي أقوم بها مفروضة علي	2
الناس الذين اهتم بهم وأحبهم يهتمون بي ويحبونني	3
أنا شخص غير مرغوب من الجماعة التي أرغب أن أنتمي إليها	4
لدي الثقة والكفاءة لإنجاز الأعمال والمهام التي تتطلبها حياتي	5
لدي شك كبير في قدراتي على القيام بالأعمال بكفاءة	6
قراراتي تنبع من إرادتي أنا وليست مما يريده مني الآخرون	7
أشعر بأنني مجبر على القيام بأعمال لا أرغب القيام بها	8
علاقتي طيبة بالناس الذين أحبهم والناس الذين يحبونني 9	9
الناس المهمين بالنسبة لي لا يعيرونني اهتمام ويبتعدون عني	10
أنا متمكن وكفؤ في ما أقوم به من أعمال	11
أنا غير راض عم مستوى كفائتي وقدراتي	12
الخيارات التي أتخذها في حياتي تعبر عني وعن شخصيتي	13
أتعرض لضغوط للقيام بأمور كثيرة في حياتي رغما عني	14
علاقاتي قوية وطيبة بالناس المهمين بالنسبة لي	15
أعتقد بأن الناس الذين أقضي الوقت معهم لا يحبونني	16
لدي القدرة والكفاءة على تحقيق أهدافي في الحياة	17
أشك في قدراتي ولا أثق بها	18
ما أقوم به في حياتي اليومية من عمل أو دراسة أحبه واعتبره مهما	19
ما أقوم به في حياتي اليومية عبارة عن سلسلة من الأمور المفروضة علي	20
أحب أصدقائي وزملائي الذين أقضي أوقاتا طويلة معهم	21
علاقاتي بالناس سطحية وليست عميقة	22
لدي الكفاءة والقدرة على إنجاز المهام والأعمال الصعبة	23
يراودني الشعور بأنني فاشل بسبب كثرة الأخطاء التي أرتكبها	24

**Table:**

العبارات	المقاييس الفرعية
1, 7, 13, 19	الرضا عن الحرية والاستقلالية
2, 8, 14, 20	عدم الرضا عن الحرية والاستقلالية
3, 9, 15, 21	الرضا عن العلاقات الاجتماعية
4, 10, 16, 22	عدم الرضا عن العلاقات الاجتماعية
5, 11, 17, 23	الرضا عن الكفاءة والقدرات
6, 12, 18, 24	عدم الرضا عن الكفاءة والقدرات

## Abbreviations

BPNSFS: Basic Psychological Need Satisfaction and Frustration Scale
BPNT: Basic Psychological Needs Theory
WEMWBS: Warwick-Edinburgh Mental Well-Being Scale
BDI-II: Beck Depression Inventory-II
CFI: Comparative Fit Index
TLI: Tucker-Lewis Index
RMSEA: Root Mean-Square Error of Approximation
BIC: Baysian Information Criterion

## Availability of the data and materials

The datasets used and/or analyzed during the current study are available anytime from the corresponding author or from the second author on reasonable request.
