# Correction to: Psychometric properties of the Arabic version of the Basic Psychological Needs Satisfaction-Frustration Scale (BPNSFS)

**DOI:** 10.1186/s40359-021-00540-7

**Published:** 2021-05-10

**Authors:** Kashef N. Zayed, Ehab N. Omara, Nasser Y. al-Rawahi, Ali K. al-Shamli, Asama A. al-Atiyah, Ahmad A. al-Haramleh, Mahmoud S. Azab, Ghada M. al-Khasawneh, Mohammed A. Hassan

**Affiliations:** 1 Sultan Qaboos University, Muscat, Oman; 2 Qatar University, Doha, Qatar; 3 Suhar University, Sohar, Oman; 4 Hafr Al-Batin University, Hafar Al Batin, Kingdom of Saudi Arabia; 5 Yarmouk University, Irbid, Jordan; 6 Palestine Technical University, Kadoorie, Tulkarm, Palestine; 7 Zagazig University, Zagazig, Egypt

## Correction to: BMC Psychol (2021) 9:15 https://doi.org/10.1186/s40359-020-00506-1

Following publication of the original article [1], the authors flagged that the article had published with incomplete affiliation details and with the name of the fifth author misspelled as ‘Asama K. al-Attiyah’ rather than ‘Asama A. al-Atiyah’.

The original article has since been updated and the corrected affiliations details and author list can be found in this correction article.
