# Supplementary material for: Psychometric properties of the Arabic version of the Basic Psychological Needs Satisfaction-Frustration Scale (BPNSFS)
Source: BMC Psychol. 2021 Jan 26;9:15. doi: 10.1186/s40359-020-00506-1 (PMC7836576; doi:10.1186/s40359-020-00506-1)
Supplement: Supplementary file 1 — Additional file 1. The Basic Psychological Need Satisfaction-Frustration Scale (BPNSFS). [file 40359_2020_506_MOESM1_ESM.pdf]

3.1.2. English version

3.1.2.1. Adults

Below, we ask you about the kind of experiences you actually have in your life. Please read each of the following items carefully. You can choose from 1 to 5 to indicate the degree to which the statement is true for you at this point in your life.

1<br>Not true at all	2	3	4	5<br>Completely true

1.	I feel a sense of choice and freedom in the things I undertake.	1	2	3	4	5
2.	Most of the things I do feel like "I have to".	1	2	3	4	5
3.	I feel that the people I care about also care about me.	1	2	3	4	5
4.	I feel excluded from the group I want to belong to.	1	2	3	4	5
5.	I feel confident that I can do things well.	1	2	3	4	5
6.	I have serious doubts about whether I can do things well.	1	2	3	4	5
7.	I feel that my decisions reflect what I really want.	1	2	3	4	5
8.	I feel forced to do many things I wouldn't choose to do.	1	2	3	4	5
9.	I feel connected with people who care for me, and for whom I care.	1	2	3	4	5
10.	I feel that people who are important to me are cold and distant towards me.	1	2	3	4	5
11.	I feel capable at what I do.	1	2	3	4	5
12.	I feel disappointed with many of my performances.	1	2	3	4	5
13.	I feel my choices express who I really am.	1	2	3	4	5
14.	I feel pressured to do too many things.	1	2	3	4	5
15.	I feel close and connected with other people who are important to me.	1	2	3	4	5
16.	I have the impression that people I spend time with dislike me.	1	2	3	4	5

17.	I feel competent to achieve my goals.	**1**	**2**	**3**	**4**	**5**
18.	I feel insecure about my abilities.	**1**	**2**	**3**	**4**	**5**
19.	I feel I have been doing what really interests me.	**1**	**2**	**3**	**4**	**5**
20.	My daily activities feel like a chain of obligations.	**1**	**2**	**3**	**4**	**5**
21.	I experience a warm feeling with the people I spend time with.	**1**	**2**	**3**	**4**	**5**
22.	I feel the relationships I have are just superficial.	**1**	**2**	**3**	**4**	**5**
23.	I feel I can successfully complete difficult tasks.	**1**	**2**	**3**	**4**	**5**
24.	I feel like a failure because of the mistakes I make.	**1**	**2**	**3**	**4**	**5**

Scoring information:

Autonomy satisfaction: items 1, 7, 13, 19
Autonomy frustration: items 2, 8, 14, 20
Relatedness satisfaction: items 3, 9, 15, 21
Relatedness frustration: items 4, 10, 16, 22
Competence satisfaction: items 5, 11, 17, 23
Competence frustration: items 6, 12, 18, 24

Supportive references:

Chen, B., Van Assche, J., Vansteenkiste, M., Soenens, B. & Beyers, W. (2015). Does psychological need satisfaction matter when environmental or financial safety are at risk? *Journal of Happiness Studies, 16,* 745-766.

Chen, B., Vansteenkiste, M., Beyers, W., Boone, L., Deci, E. L., Van der Kaap-Deeder, J., Duriez, B. Lens, W., Matos, L., Mouratidis, A., Ryan, R. M., Sheldon, K. M., Soenens, B., Van Petegem, S., & Verstuyf, J. (2015). Basic psychological need satisfaction, need frustration, and need strength across four cultures. *Motivation and Emotion, 39,* 216-236.

Contact person:

Maarten Vansteenkiste; Maarten.Vansteenkiste@UGent.be.
