# Supplementary material for: Psychometric properties of the Arabic version of the Basic Psychological Needs Satisfaction-Frustration Scale (BPNSFS)
Source: BMC Psychol. 2021 Jan 26;9:15. doi: 10.1186/s40359-020-00506-1 (PMC7836576; doi:10.1186/s40359-020-00506-1)
Supplement: Supplementary file 2 — Additional file 2. The Warwick-Edinburgh Mental Well-Being Scale (WEMWBS). [file 40359_2020_506_MOESM2_ESM.pdf]

The Warwick–Edinburgh Mental Well-being Scale (WEMWBS)

Below are some statements about feelings and thoughts.

Please tick the box that best describes your experience of each over the last 2 weeks

STATEMENTS	None of the time	Rarely	Some of the time	Often	All of the time
I’ve been feeling optimistic about the future	1	2	3	4	5
I’ve been feeling useful	1	2	3	4	5
I’ve been feeling relaxed	1	2	3	4	5
I’ve been feeling interested in other people	1	2	3	4	5
I’ve had energy to spare	1	2	3	4	5
I’ve been dealing with problems well	1	2	3	4	5
I’ve been thinking clearly	1	2	3	4	5
I’ve been feeling good about myself	1	2	3	4	5
I’ve been feeling close to other people	1	2	3	4	5
I’ve been feeling confident	1	2	3	4	5
I’ve been able to make up my own mind about things	1	2	3	4	5
I’ve been feeling loved	1	2	3	4	5
I’ve been interested in new things	1	2	3	4	5
I’ve been feeling cheerful	1	2	3	4	5

Warwick–Edinburgh Mental Well-being Scale (WEMWBS)
© NHS Health Scotland, University of Warwick and University of Edinburgh, 2006, all rights reserved.
